# Deployment of Infectious Disease Experts and Prevalence of Antimicrobial Resistance in Okayama: A Call for Training of Specialists

**DOI:** 10.7759/cureus.16643

**Published:** 2021-07-26

**Authors:** Hideharu Hagiya, Koji Fujita, Shinya Kamiyama, Kazuki Ocho, Haruto Yamada, Fumio Otsuka

**Affiliations:** 1 Department of General Medicine, Okayama University Graduate School of Medicine, Dentistry and Pharmaceutical Sciences, Okayama, JPN; 2 Department of General Medicine and Infectious Disease, Tsuyama Chuo Hospital, Tsuyama, JPN; 3 Department of Laboratory Medicine and Infectious Diseases, Kurashiki Central Hospital, Kurashiki, JPN; 4 Department of Clinical Laboratory, Okayama City Hospital, Okayama, JPN

**Keywords:** antimicrobial resistance, certified nurses in infection control, infection prevention and control, infectious disease doctors, japan nosocomial infections surveillance

## Abstract

Objective

During the ongoing global pandemic of novel coronavirus disease 2019 (COVID-19), an emerging infectious disease, the implementation and execution of infection prevention and control (IPC) is of paramount importance. In this study, we aimed to assess the current deployment of infection control medical personnel in Okayama prefecture, who are supposed to play an essential role to prevent the outbreak of infectious diseases, and the current prevalence of antimicrobial-resistant (AMR) bacteria isolated in Okayama.

Materials and methods

This was a descriptive study using publicly available data. The numbers of infectious disease (ID)-doctors and the certified nurses in infection control (CNIC) per 100,000 population in 47 prefectures in Japan were calculated. We then compared the detected proportions of AMR pathogens among the prefectures in 2019 to be employed as a comparative parameter, which was obtained from Japan Nosocomial Infections Surveillance (JANIS) data.

Results

The number of ID-doctors was the 11th^ ^highest in Japan; however, they were unevenly distributed in southern Okayama, particularly at three tertiary hospitals. While the deployment of CNIC was geographically less uneven in the prefecture, their number was lower than the domestic average. According to the JANIS data, isolation rates of AMR pathogens were high in Okayama compared to other prefectures in Japan: vancomycin-resistant *Enterococcus faecium* (the third-worst); cefotaxime-resistant *Escherichia coli* and *Klebsiella pneumoniae* (the third-worst and the second-worst, respectively); and meropenem-resistant *Pseudomonas aeruginosa* (the worst).

Conclusions

Our assessment provides underlying data and reinforces the need for educating multi-professional experts in the field of infectious diseases to prevent future public health threats in Okayama.

## Introduction

The current global pandemic of novel coronavirus disease 2019 (COVID-19) has strongly raised social awareness concerning the importance of infection prevention and control (IPC) in medical institutions and communities [1]. In response to the unprecedented pandemic, we progressively need to devise a framework for the IPC system based on multidisciplinary perspectives, such as medical, epidemiological, administrative, financial, legal, and economic views. It is especially essential to boost both the quality and quantity of preventive measures at medical facilities so that we can prevent nosocomial infections and provide safe healthcare to the public.

In this vertically-segmented medical specialist system, infectious disease (ID)-doctors and the certified nurses in infection control (CNIC) are expected to play an active role in implementing IPC activities at each medical institution. As of October 1, 2020, the number of Board Certified Physicians of the Japanese Association for Infectious Diseases was 1,554 in Japan [2]. A total of 2,852 registered nurses had been certified as CNIC by the Japanese Nursing Association [3]. According to a statement issued by the Japanese Association for Infectious Diseases, each medical institution with over 300 beds should have at least one full-time ID-doctor on duty [4]. For CNIC, the Centers for Disease Control and Prevention of the United States mandates that medical facilities should have one CNIC per 250 hospital beds [5]. As for IPC services in clinical settings, it is not realistic to uniformly deploy or centralize the expertise by establishing a specific infectious disease center, because the infectious diseases are diverse and numerous, and usually develop acutely and require a rapid response. Hence, it is now generally accepted that medical facilities of a certain size should have ID-doctors and CNIC on-site in full-time positions to develop highly effective IPC activities.

In this study, we aimed to determine the current deployment status of ID-doctors and CNIC in Okayama prefecture based on quantitative and geographical perspectives, in comparison to the rest of Japan. Additionally, we assessed the recent prevalence of antimicrobial-resistant (AMR) bacteria isolated in Okayama by searching the national open data provided by the Japanese Ministry of Health, Labor, and Welfare. Our main goal is to provide fundamental data to educate multi-professional experts in the field of infectious diseases in Okayama.

## Materials and methods

This was a descriptive study that used publicly available data. Population data were collected from the website of the Statistics Bureau of the Ministry of Internal Affairs and Communications [6]. We defined ID-doctors as those who are registered as Board Certified Physicians of the Japanese Association for Infectious Diseases. Data on the number of ID-doctors at each prefecture, as of October 1, 2020, was obtained from the Japanese Association for Infectious Diseases [2]. Additionally, we defined CNIC accredited by the Japanese Nursing Association as those who are employed as infection control nurses at medical facilities [3]. We calculated the numbers of ID-doctors and CNIC per 100,000 population in each prefecture and comparisons were performed among the prefectures. To understand the geographical distribution of ID-doctors and CNIC in Okayama prefecture, we divided the Okayama prefecture into three distinct regions: (i) a south-eastern region around Okayama city, (ii) a south-western region around Kurashiki city, and (iii) a northern region around Tsuyama city.

To evaluate the effectiveness of IPC activities in Okayama, we compared the detected proportions of AMR pathogens among the prefectures to be employed as a comparative parameter. Data on the isolation rates of methicillin-resistant *Staphylococcus aureus* (MRSA), vancomycin (VCM)-resistant *Enterococcus faecium* (VRE), third-generation cephalosporin [cefotaxime (CTX)]-resistant *Escherichia coli (E. coli) *and* Klebsiella pneumoniae (K. pneumoniae)*, fluoroquinolone [levofloxacin (LVFX)]-resistant *E. coli* and *K. pneumoniae*, and carbapenem [meropenem (MEPM)]-resistant *Pseudomonas aeruginosa (P. aeruginosa)* were collected from Japan Nosocomial Infections Surveillance (JANIS) data, which was open to the public [7]. The JANIS service was launched in 2000 as a sustainable surveillance model for national AMR data with the support of the Japanese government, which has been developed into one of the largest national AMR surveillance worldwide [8,9]. MRSA is generally defined as an *S. aureus* strain showing resistance to either oxacillin or cefoxitin (CFX); however, in this study, no corresponding data were available in the JANIS public information system, and hence we considered CFX-resistant *S. aureus* strain as MRSA for the purpose of this study.

No statistical analysis was performed in this study. Ethics committee approval and the need for informed consent were waived off by the Institutional Review Board of the Okayama University Hospital since the detailed data on individuals and hospitals were all anonymized (No. 2103-042).

## Results

**Figure 1:**
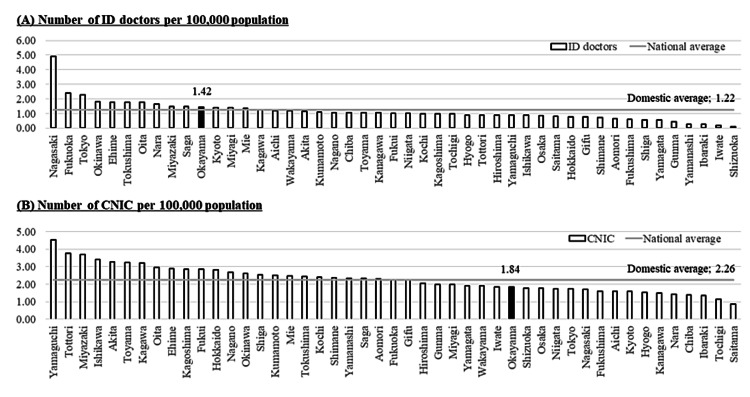
The number of ID-doctors (A) and CNIC (B) per 100,000 population by prefectures and the national average in Japan ID: infectious diseases; CNIC: certified nurse in infection control

The number of ID-doctors in Okayama was 27, which amounts to 1.42/100,000 population and is the 11th highest in Japan (the domestic average is 1.22/100,000 population) (Figure 1A). The number of CNIC in Okayama was 35 (corresponding to 1.84/100,000 population), which was lower than the domestic average of 2.26/100,000 population (Figure 1B).

Geographical deployment of ID-doctors and CNIC in the Okayama prefecture

**Figure 2:**
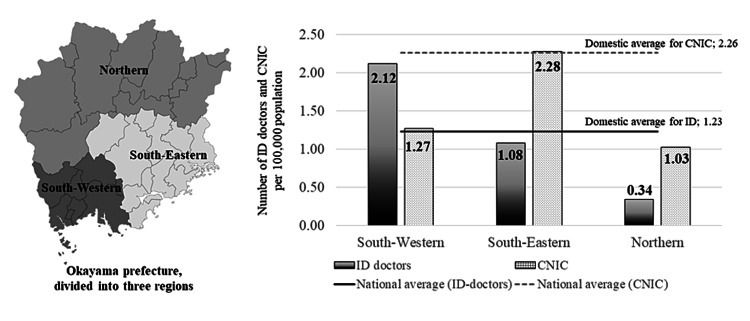
The number of ID-doctors and CNIC per 100,000 population in three distinct regions of the Okayama prefecture and the national average ID: infectious diseases; CNIC: certified nurse in infection control

We have presented the number of ID-doctors and CNIC per 100,000 population in three distinct regions of Okayama prefectures along with the domestic average (Figure 2). Out of 27 ID-doctors, we used the affiliation data of 26 ID-doctors because the data of one doctor was not available. We included the data of 32 CNIC in the investigation by excluding three nurses (one did not belong to a medical institution and the affiliation data of two were unknown). In the south-eastern region, there were 10 ID-doctors in Okayama city (but not in the other cities) and 20 CNIC (16 in Okayama city, three in Setouchi city, and one in Kibi Chuo town). The population ratios of ID-doctors (1.08/100,000 population) and CNIC (2.28/100,000 population) were nearly equal to the domestic averages. In the south-western region, there were 15 ID-doctors (13 in Kurashiki city, one in Kasaoka city, and one in Hayashima town) and nine CNIC (seven in Kurashiki city, one in Ibara city, and one in Hayashima town). The population ratio of ID-doctors (2.12/100,000 population) was well above the domestic average (1.23/100,000 population), while that of CNIC in the region (1.27/100,000 population) was below the domestic average (2.26/100,000 population). In the northern region, there was one ID-doctor (Mimasaka city) and three CNIC (two in Tsuyama city and one in Maniwa city). The population ratios of ID-doctors (0.34/100,000 population) and CNIC (1.03/100,000 population) were much below the domestic averages. Deployment of ID-doctors in the south-eastern and south-western regions seems sufficient; however, when studied in detail, we found that their affiliations/distribution were uneven, and they were mostly found affiliated to two university hospitals (seven at Kawasaki Medical School Hospital and four at Okayama University Hospital) and a tertiary hospital (four at Kurashiki Central Hospital). In contrast, we found that CNIC was almost evenly distributed in the major medical institutions in each region.

Recent epidemiological data on AMR bacteria in Okayama in comparison with other prefectures

The number of medical institutions in Okayama that provided microbiology data to JANIS in the fiscal year 2019 was 50 in total; two of them had more than 900 beds, four had 500-899 beds, 13 had 200-499 beds, and 31 had less than 200 beds. This corresponded to 30.7% of medical institutions registered in the prefecture.

**Figure 3:**
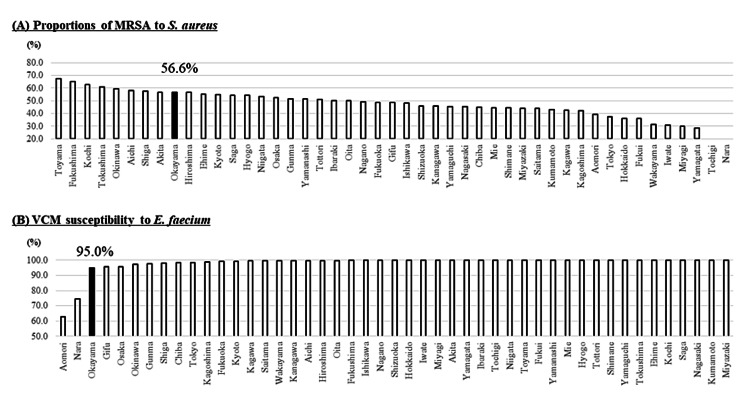
The proportion of MRSA to the total isolation of S. aureus (A) and antimicrobial susceptibility of VCM for E. faecium (B) in each prefecture of Japan in 2019 The detection rate of the AMR bacteria increases from the right to the left. The proportion of MRSA was estimated by cefoxitin susceptibility testing MRSA: methicillin-resistant *Staphylococcus aureus; S. aureus: Staphylococcus aureus; *VCM: vancomycin; *E. faecium: Enterococcus faecium; *AMR: antimicrobial-resistant

**Figure 4:**
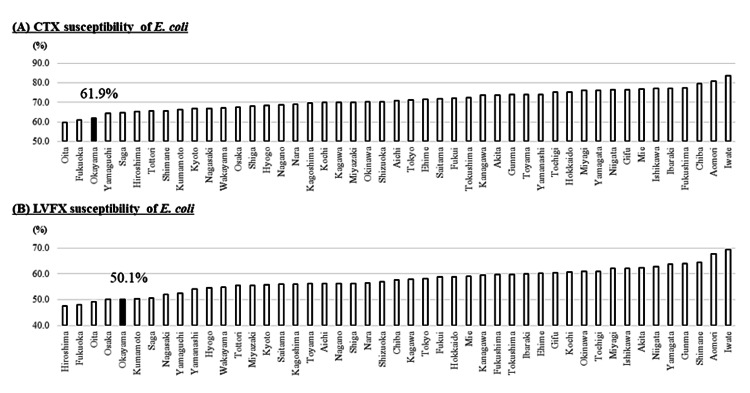
Antimicrobial susceptibility rates of CTX (A) and LVFX (B) for E. coli in each prefecture of Japan in 2019 The detection rate of the AMR bacteria increases from the right to the left CTX: cefotaxime; LVFX: levofloxacin; *E. coli: Escherichia coli*; AMR: antimicrobial-resistant

**Figure 5:**
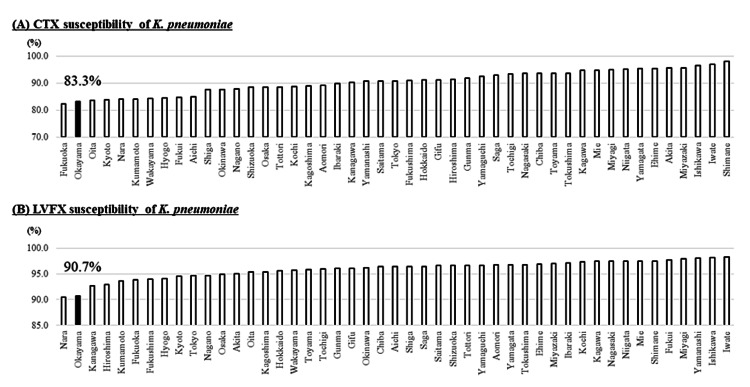
Antimicrobial susceptibility rates of CTX (A) and LVFX (B) for K. pneumoniae in each prefecture of Japan in 2019 The detection rate of the AMR bacteria increases from the right to the left CTX: cefotaxime; LVFX: levofloxacin; *K. pneumoniae: Klebsiella pneumoniae; *AMR: antimicrobial-resistant

**Figure 6:**
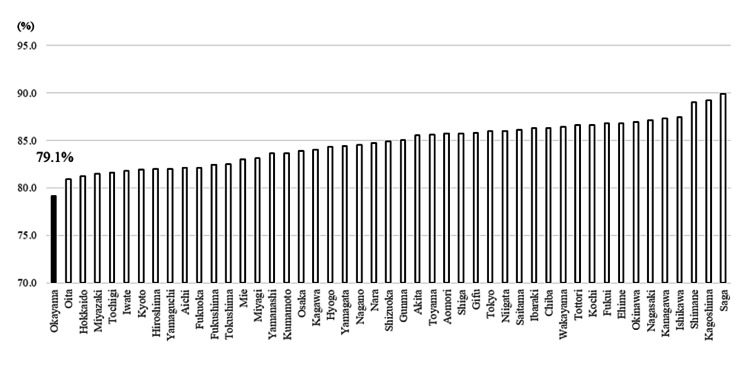
Antimicrobial susceptibility rates of meropenem for P. aeruginosa in each prefecture of Japan in 2019 The detection rate of the AMR bacteria increases from the right to the left *P. aeruginosa: Pseudomonas aeruginosa;* AMR: antimicrobial-resistant

Isolation rates of MRSA were 56.6% (the 9th most prevalent) (Figure 3A). VCM susceptibility rates of *E. faecium* were 95.0% (the 3rd lowest), i.e., isolation rates of VRE in *E. faecium* were 5.0% (Figure 3B). CTX- and LVFX-susceptibility rates of *E. coli* were 61.9% (the 3rd lowest) and 50.1% (the 5th lowest), respectively (Figure 4). Similarly, CTX- and LVFX-susceptibility rates of *K. pneumoniae* were 83.3% (the 2nd lowest) and 90.7% (the 2nd lowest), respectively (Figure 5). Finally, MEPM-susceptibility rates of *P. aeruginosa* were 79.1% (the lowest) (Figure 6).

## Discussion

In this study, we evaluated the current deployment status of infectious diseases experts in Okayama prefecture based on publicly available data and compared it with that of other prefectures. Briefly, although the number of ID-doctors in Okayama was the 11th highest among the prefectures in Japan, they were unevenly distributed and a higher number of ID-doctors were present in the southern regions, particularly at three tertiary hospitals. There were no ID-doctors at a few of the key regional hospitals. In the northern region, ID-doctors were underrepresented in terms of numbers. In contrast, in the case of CNIC, there was a less geographically biased distribution; however, the registered number in Okayama was comparatively small. Notably, based on the JANIS data, the isolation rates of AMR pathogens in Okayama were high in the year 2019. MRSA, VRE, and CTX- and LVFX-resistant *E. coli* and* K. pneumoniae*, and carbapenem-resistant *P. aeruginosa* are all representative nosocomial pathogens that should be brought under control by strict IPC activities. Because of its sophisticated system and well-established database, the JANIS data is reported to be a powerful surveillance tool for the regional prevalence of AMR pathogens [10] and has the potential to be further applied for automated AMR detection systems by using a cluster detection software tool, called SaTScan [11]. Thus, JANIS data is useful to understand the trends in the detection of AMR pathogens. High detection rates of the representative AMR organisms may suggest a relative inadequacy of IPC measures in Okayama, which might be, at least partially, a consequence of the shortage and unequal distribution of the infectious diseases expertise.

Although the number of ID-doctors appears to be sufficient, their skewed distribution in the prefecture limits the promotion of IPC activities across the prefecture. Besides, the ID-doctors would have diverse clinical backgrounds and subspecialties, and thus, their knowledge and experiences in IPC greatly differ from each other. It is also true that non-ID-doctors are responsible for the IPC activities at each facility and function effectively. Thus, it would be not enough to evaluate the activity level of IPC in the whole prefecture solely based on the distribution of ID-doctors, which could be one of the limitations of this study. Other qualifications that potentially cover the ability of IPC for physicians include the Infection Control Doctor (ICD) system, Certified Board of Pediatric Infectious Diseases (accredited by the Japanese Society for Pediatric Infectious Diseases), Fellow of Japanese Antimicrobial Chemotherapy Physician (accredited by the Japanese Society of Chemotherapy), and Certified Fellow of the Japanese Society for Clinical Microbiology. However, none of them can assure the doctor's ability to contribute to the IPC. As of January 2019, the number of ICD holders reached 9,362 in the whole country, but no data by prefectures are open to the public and, hence, we are skeptical as to the number of individuals among these ICDs who have actually contributed to the IPC activity at their institutions.

In contrast with ID-doctors, there was no geographic imbalance in CNIC deployment, although the number of CNIC was comparatively less. A plausible reason for the absence of uneven distribution in CNIC could be that a full-time CNIC on duty is a requirement for obtaining additional reimbursement for infection prevention. In other words, the education and employment of CNIC can directly increase the revenue for healthcare facilities. Another limitation of this study is that it is unclear whether CNIC certificate holders work full-time in such positions at their respective medical institutions. That is, although the registered number of CNIC certificate holders appears to be sufficient, some of them may not necessarily be working as CNIC. Similar to medical doctors, there are other career paths or certifications other than CNIC in the nursing profession, and the assessment for CNIC alone may be insufficient to estimate the current sufficiency of nurses involved in IPC activities in Okayama. To get certified as CNIC, a nurse has to join a half-year training course and spend several million Japanese yen at personal capacity. The time and economic constraints have surely hindered the efforts to increase the number of CNIC certificate holders. To further ensure that medical institutions in Okayama are well equipped with CNIC, flexible employment and financial support for their education would be indispensable.

To evaluate the activity of IPC, antimicrobial stewardship should be a good comparative parameter as well. In the Japanese National Action Plan on Antimicrobial Resistance launched in 2016 [12], compared to 2013, it was proposed that the use of antimicrobials in 2020 should be reduced by 33% in total: 50% for oral cephalosporins, fluoroquinolones, and macrolides, and 20% for intravenous drugs. Based on the National Database of Health Insurance Claims and Specific Health Checkups of Japan, calculated based on the data from AMR Clinical Reference Center [13], as of 2019, the use of intravenous agents increased by 3.9% across the country. However, the oral antimicrobial use of cephalosporins, quinolones, and macrolides favorably decreased by 14.2%, 9.7%, and 12.7%, respectively, although the goals were not achieved till 2020. In addition, according to the AWaRe classification proposed by the World Health Organization (WHO), a proportion of Access group antimicrobials is on an increasing trend (18.1%, as of 2019), while that of Watch group antimicrobials has decreased to 80.1%, suggesting that the antimicrobial use is gradually being optimized in Japan [14]. However, the data from AMR Clinical Reference Center revealed that the defined daily dose of intravenous/oral and oral antimicrobial uses in Okayama was 14.95 (the 11th highest in the country) and 13.93 (the 12th highest in the country), respectively [15]. This data indicates the need to further promote antimicrobial stewardship in Okayama, besides IPC activities. From a long-term perspective, in addition to continuous education to medical doctors, it is also fundamental to use the bottom-up approach to increase awareness and knowledge of the proper use of antimicrobials in pre- and post-graduate educational systems.

Not only the medical doctors and nurses, but also other medical practitioners such as pharmacists, clinical laboratory technicians, and even clerical staff could play an important role in IPC activities. For example, pharmacists should be closely involved in antimicrobial stewardship, or the appropriate use of antimicrobial drugs. Clinical laboratory technicians should make a significant contribution to the rapid detection and data management of AMR bacteria. Professional qualifications systems for the medical personnel, namely, infectious disease chemotherapy pharmacist (IDCP) and board-certified infection control pharmacy specialist (BCIPS) for pharmacists, and infection control microbiological technologist (ICMT) for clinical laboratory technicians, have already been established. Professional education in these fields could further facilitate the IPC activities in a hospital.

## Conclusions

Our analysis revealed the uneven distribution of ID-doctors and a shortage of CNIC in Okayama, despite the high isolation rates of AMR pathogens. Although the causal relationship between these data is unclear, we should progressively work on improving the current situation. First of all, from a long-term perspective, medical schools need to include clinical infectious diseases in the pre-graduate education curriculum. Additionally, as a subject of intensive post-graduate education, infectious diseases should be more frequently and regularly addressed, which could help in the increase of ID expertise in the future. Currently, we need to establish a comprehensive system for regional cooperation with neighboring healthcare institutions in improving the level of IPC in the entire region. This is the right time to promote a continuous education system concerning infectious diseases to achieve sustainable development goals in terms of infection control and prevention in Okayama.
